# Supplementary material for: Inhibition of a K9/K36 demethylase by an H3.3 point mutation found in paediatric glioblastoma
Source: Nat Commun. 2018 Aug 7;9:3142. doi: 10.1038/s41467-018-05607-5 (PMC6081460; doi:10.1038/s41467-018-05607-5)
Supplement: Supplementary file 1 — Supplementary Information [file 41467_2018_5607_MOESM1_ESM.pdf]

Inhibition of a K9/K36 Demethylase by an H3.3 Point Mutation Found in Paediatric Glioblastoma

Voon *et al.*

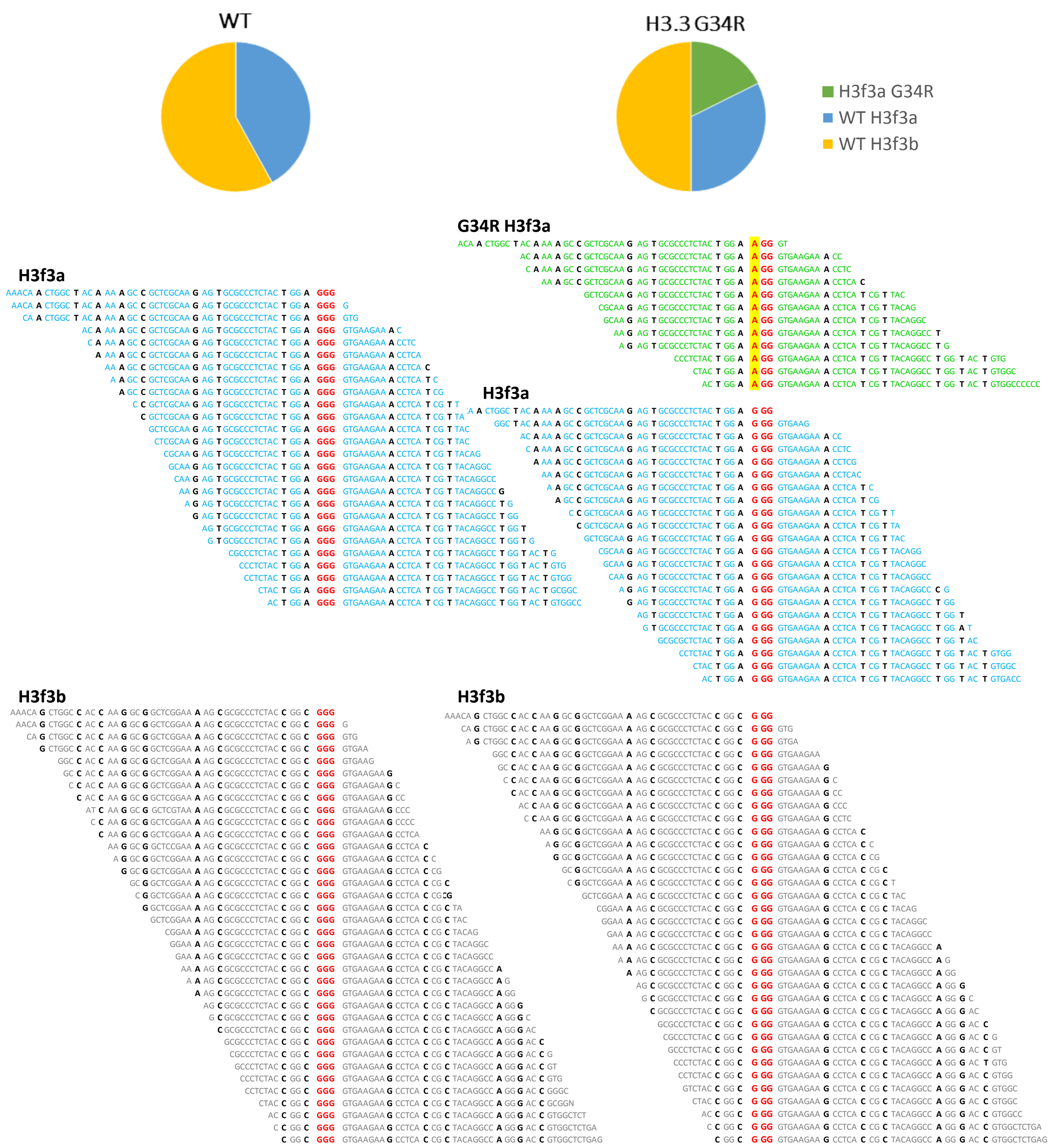

Supplementary Figure 1. RNA-sequencing reads from WT and G34R cells which span codon 34 of *H3f3a* and *H3f3b*. Green, blue and grey text indicate reads which map to *H3f3a* G34R, WT *H3f3a*, and WT H3f3b respectively. Bold black text indicate nucleotides which differentiate between *H3f3a* and *H3f3b*. Codon 34 is indicated in red and the glycine to arginine point mutation is highlighted in yellow.

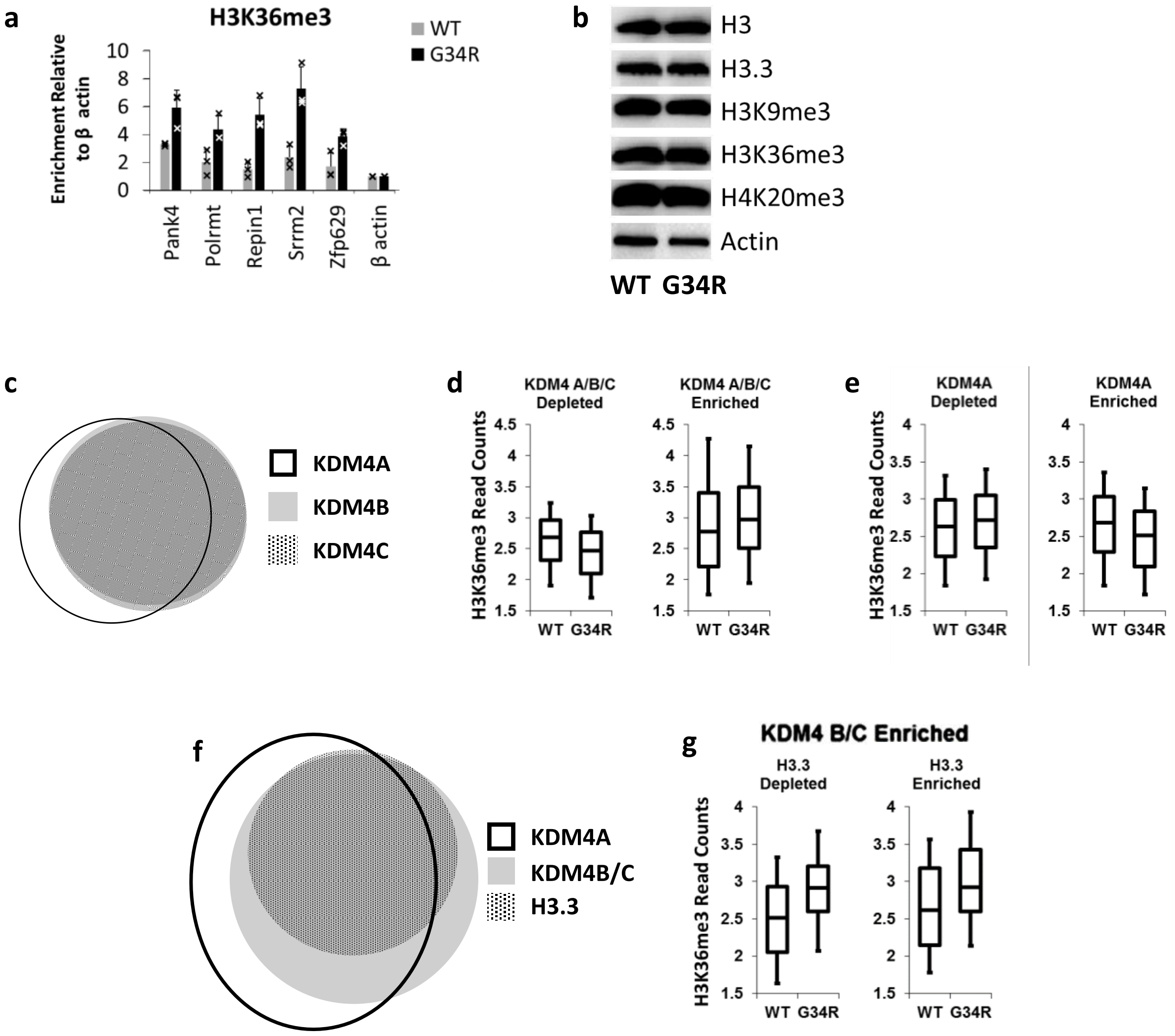

Supplementary Figure 2. (a) Dot plot overlay of H3K36me3 ChIP-qPCR in WT and G34R cells. **(b)** Western blot of whole cells extracts from WT and G34R cells. **(c)** Overlaps between KDM4 -A, -B, and -C at H3K36me3 enriched genomic regions. **(d)** Normalised H3K36me3 read counts from WT and G34R cells at KDM4 A/B/C depleted (*n* = 1957) and enriched (*n* = 2596) genomic regions. **(e)** Normalised H3K36me3 read counts from WT and G34R cells at KDM4A depleted (*n* = 8909) and enriched (*n* = 778) genomic regions. **(f)** Overlaps between KDM4A, KDM4 B/C, and H3.3 at H3K36me3 enriched genomic regions. **(g)** Normalised H3K36me3 read counts from WT and G34R cells at KDM4 B/C enriched sites which are depleted (*n* = 189) or enriched (*n* = 120) for H3.3. Boxes represent 25th, median and 75th percentile; whiskers represent 10th and 90th percentiles.

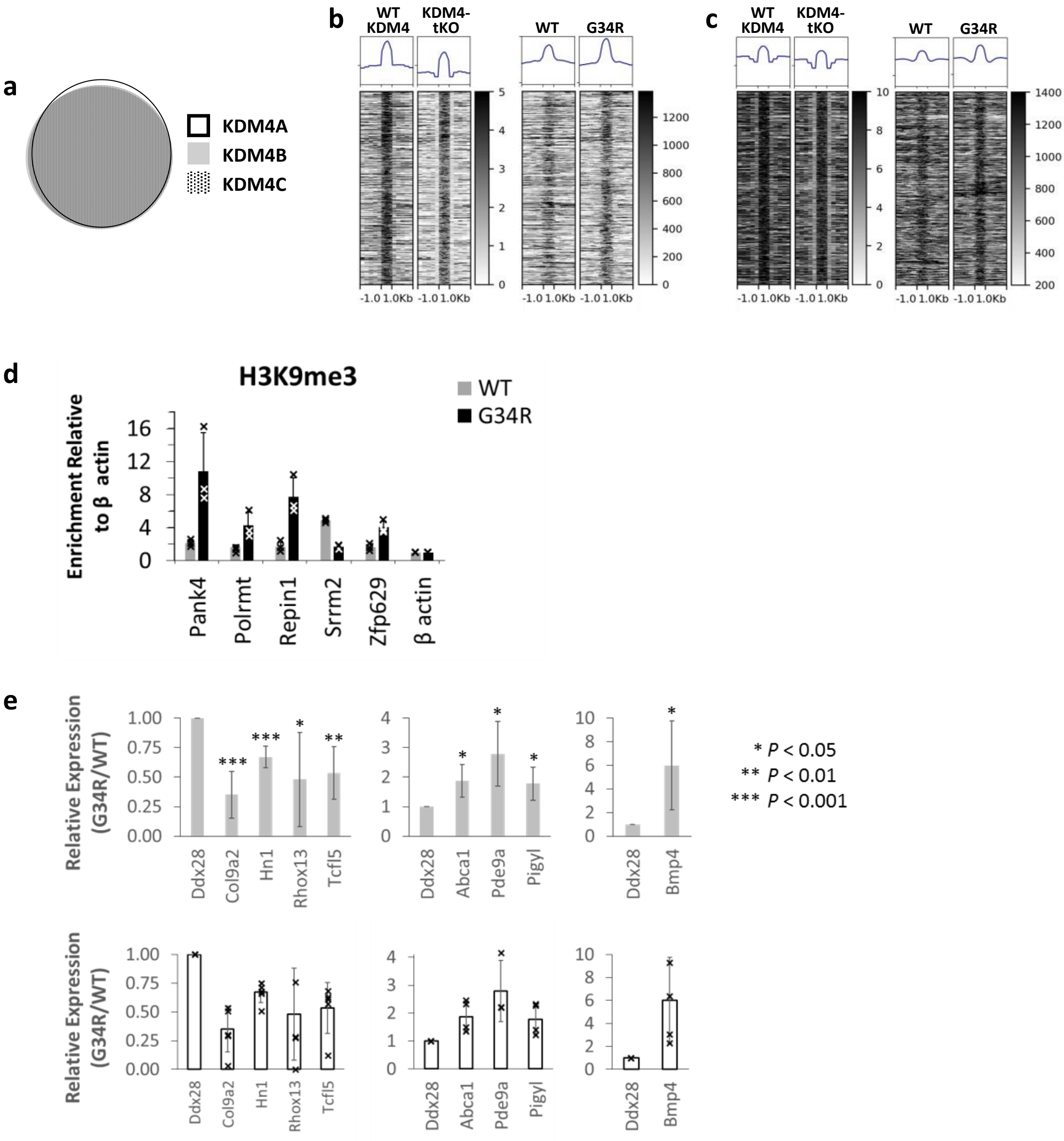

Supplementary Figure 3. (a) Overlaps between KDM4 -A, -B, and -C at H3K9me3 enriched genomic regions. **(b-c)** Heatmaps of **(b)** H3K9me3 (n = 932) and **(c)** H3K36me3 (n = 5136) ChIP-seq reads at sites where H3K9me3 or H3K36me3 is unchanged (0.8 - 1 fold) in KDM4-tKO mutants (+/- 1 kb) with profiles of corresponding regions in WT and G34R cells. **(d)** Dot plot overlay of H3K36me3 ChIP-qPCR in WT and G34R cells. **(e)** RT-qPCR of selected genes identified as down- or up-regulated in G34R cells relative to WT. Dot plot overlays are also shown. Bars represent mean values of G34R/WT expression calculated relative to an unchanged gene (Ddx28) (n = 3). Error bars represent standard deviation of three independent experiments (n = 3). P-values were calculated using Student's T-test (*$P < 0.05$, **$P < 0.01$, ***$P < 0.001$).

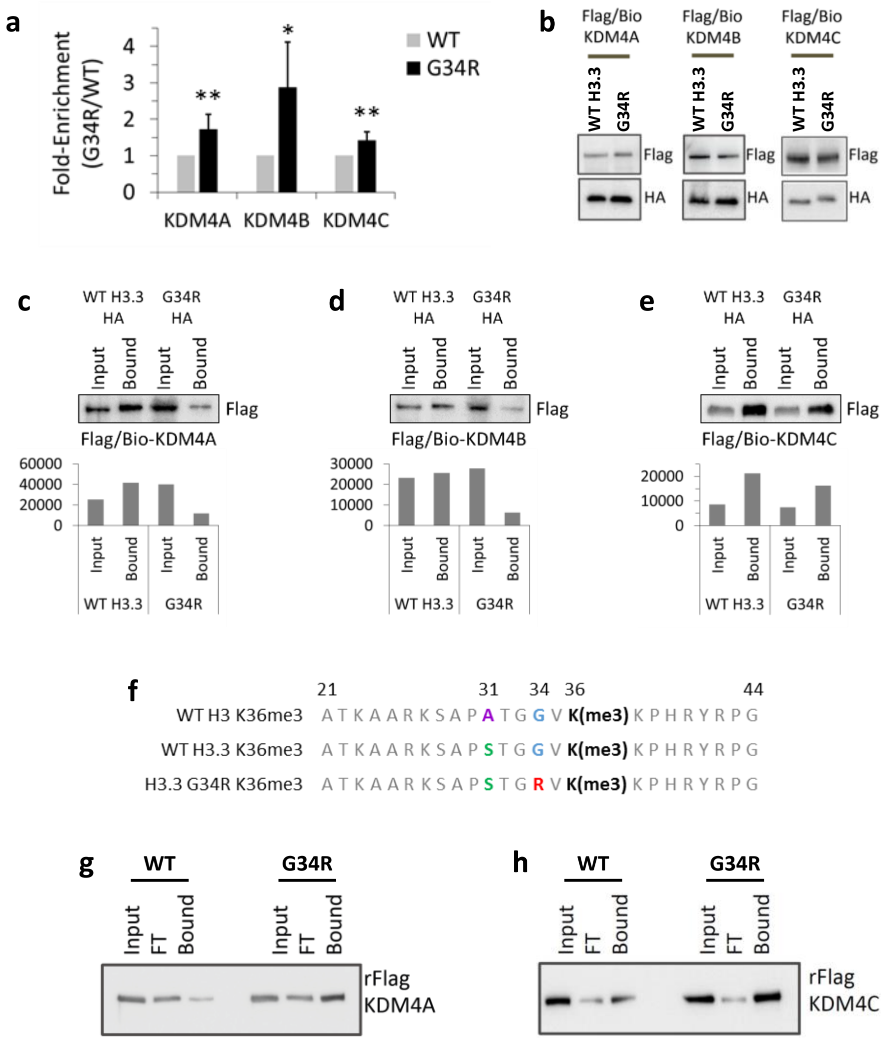

Supplementary Figure 4. (a-e) Co-transfection experiments of Flag/Biotin-tagged KDM4 -A, -B, and -C, with HA-tagged WT H3.3 or H3.3 G34R. **(a)** Quantitation of protein pulldowns of KDM4 -A -B, and -C, immunoblotted with anti-HA for detection of HA-tagged WT H3.3 and H3.3 G34R. Bars represent mean relative value of G34R/WT-H3.3 and error bars represent the standard deviation of three independent experiments ($n = 3$). P-values were calculated using Student's T-test (*$P < 0.05$, **$P < 0.0005$). **(b)** Western blots with anti-Flag and anti-HA antibodies showing expression levels of KDM4 A/B/C and WT/G34R H3.3. **(c-e)** Immunoprecipitation of **(c)** KDM4A **(d)** KDM4B and **(e)** KDM4C immunoblotted with Anti-Flag for detection of KDM4. **(f-h)** *In vitro* binding assays of Flag-tagged KDM4 -A and -C with WT H3.3 K36me3 and H3.3 G34R K36me3 peptides. **(f)** Synthetic peptides used in assay with key amino acids indicated. Pulldown of **(g)** KDM4A and **(h)** KDM4C comparing interactions between WT H3.3 K36me3 and H3.3 G34R K36me3 peptides. FT = flowthrough.

HindIII digest; Probe 1

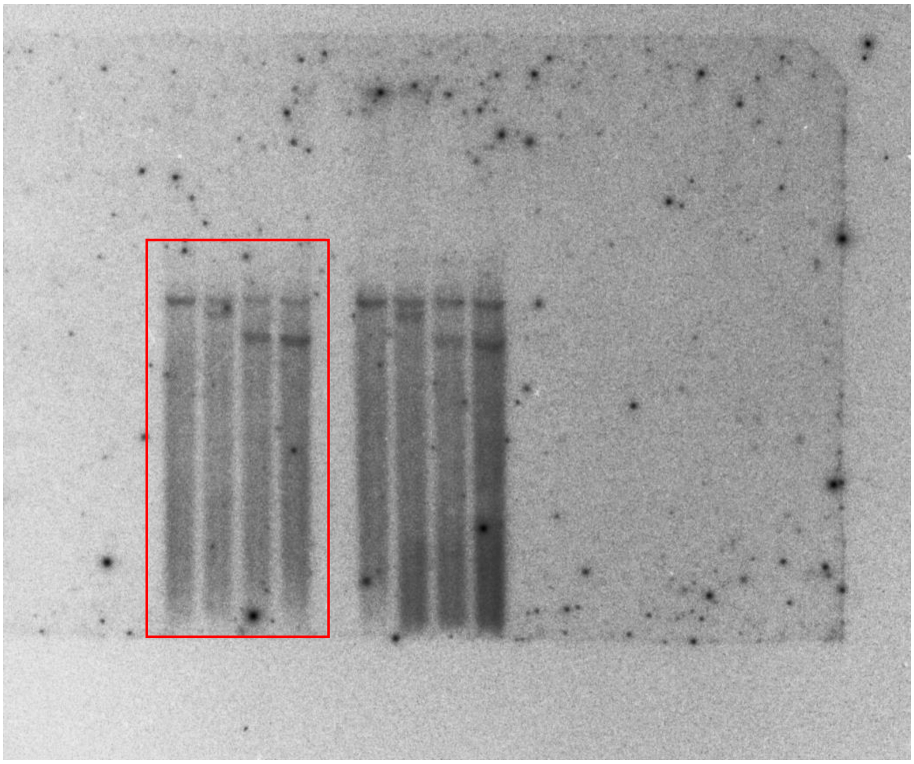

EcoRI digest; Probe 2

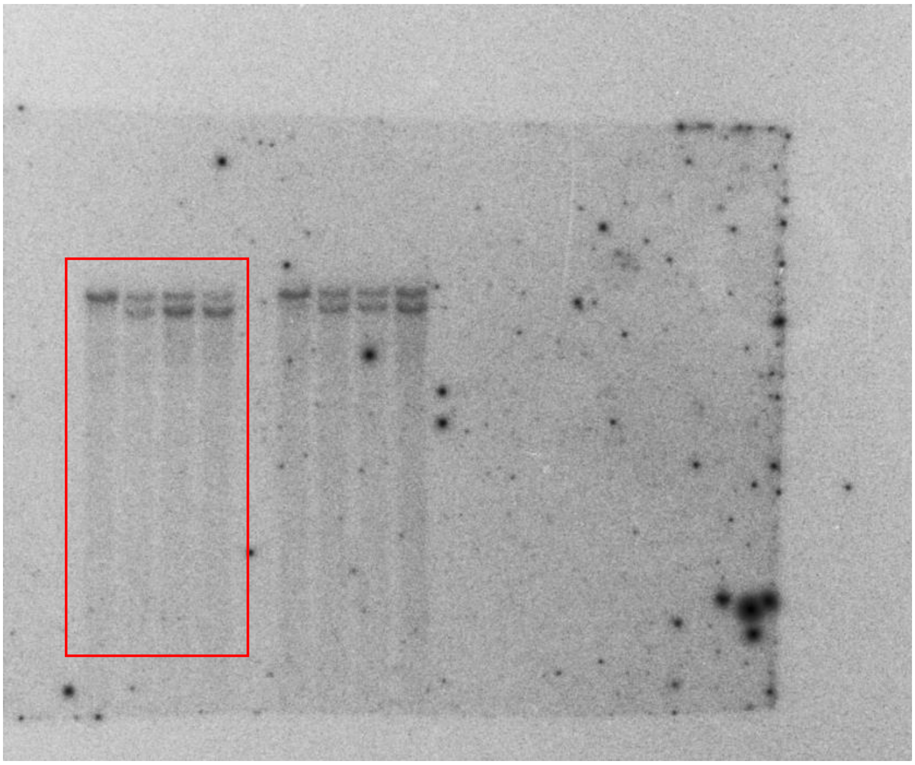

Supplementary Figure 5. Full blots of Figure 1b

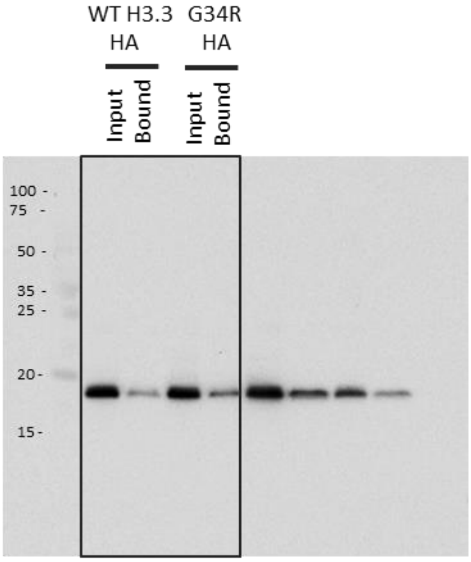

Flag/Bio-KDM4A

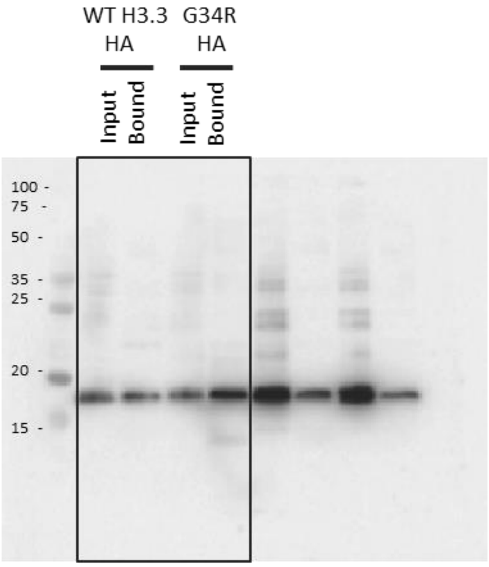

Flag/Bio-KDM4B

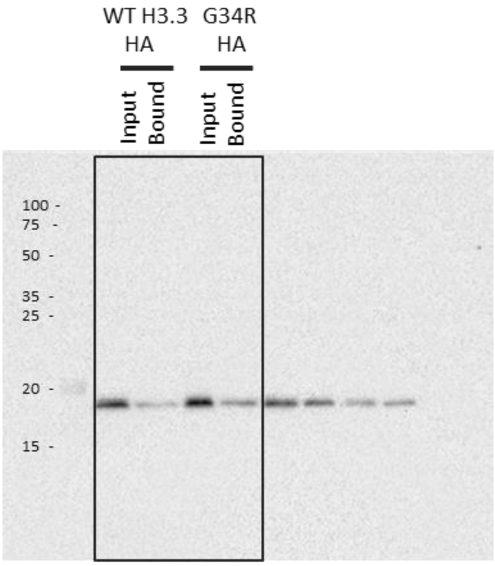

Flag/Bio-KDM4C

Supplementary Figure 6. Full blots of Figure 4a

Supplementary Table 1. List of PCR primers used in this study.

**Primers for Southern Probes**	
Probe 1 Fwd	ACCATGCTGGGCTCTTTAC
Probe 1 Rev	AACTGTGCTAGGCACAGC
Amplicon size – 295 bp	
Probe 2 Fwd	ACTAGAAAAACCGGCCAA
Probe 2 Rev	GCATATACGGAGTCAGGGA
Pank4_Rev	CTGCGTAGTTCTCACCCCAG
Amplicon size – 306 bp	
**Transgene Sequencing Primers**	
Seq_Fwd	CTCATAGTGGACTCGCGGC
Seq_Rev	GACGGCGCGTGAATTGGAT
**ChIP-qPCR Primers**	
Pank4_Fwd	TTTGAATCAAGGGCGAGGCT
Pank4_Rev	CTGCGTAGTTCTCACCCCAG
Polrmt_Fwd	CGGCCGCGGAAGTCCATGTT
Polrmt_Rev	CGCAGCGAGGCCCTGTATCG
Repin1_Fwd	GGGAAGCGTTTCACCAACAAG
Repin1_Rev	GCGACAACAGGTTGGGTTTG
Srrm2_Fwd	CCTACCAAAGGTTCTCGGCA
Srrm2_Rev	AAATCTCTCGACGGGACCCA
Zfp629_Fwd	TTGGCTCCTGGTGGCGGAGT
Zfp629_Rev	GCCCGGAGAGCTCAGGGTGA
β-actin_Fwd	ACACCCGCCACCAGGTAAGCA
β-actin_Rev	CCTGCAGTGAGGTACTAGCCACGA
**Zfp358 primers arranged 5’-3’**	
Zfp358_1_Fwd	AGCAACATCCAGAGTCCAGC
Zfp358_1_Rev	ACTTTAAAGCCCCAGCTCCC
Zfp358_2_Fwd	TTCCCAAGAGATGATCGGGG
Zfp358_2_Rev	GCAGCCTCATGAACTCCCAT
Zfp358_3_Fwd	CCAGTCCTCTCTCCCCAAGA
Zfp358_3_Rev	AACTGCTTGCAGCTTCTGGA
Zfp358_4_Fwd	GCAGAGTTCAGCACTCCTACAA
Zfp358_4_Rev	ACAGAGCTGACAACGGTAGG
Zfp358_5_Fwd	CCGGAAGAGTGCAGGTTTCA
Zfp358_5_Rev	CCCACACTGCTACACAGGAT
Zfp358_6_Fwd	TTGTCAGCTGCCATGTGGAT
Zfp358_6_Rev	TGCATCAGTGAGGAGAACTGG

**Expression Primers**	
Abca1_Fwd	AGGACAGTGTTTCTCAGAGCAG
Abca1_Rev	GAGACATCGATGGTCAGCGT
Bmp4_Fwd	GCCCTCGACCAGGTTCATTG
Bmp4_Rev	GAATGGCTCCATTGGTTCCTG
Col9a2_Fwd	AGGGAGTGCGGATTTCTTGT
Col9a2_Rev	TGCCTGGATGACCCTTCACT
Ddx28_Fwd	GTATTCTGCAACAGCGCCAG
Ddx28_Rev	TTTGCCCTTGCAGCCTTAGA
Hn1_Fwd	CAGGGAAGATTCGGAGTCGC
Hn1_Rev	TCACCTTCTCCCTTGAGGTCTA
Pde9a_Fwd	GTGAAGCAAGTGTCTGAACGA
Pde9a_Rev	TCGTTGATCTTAAACGCTCTGGA
Pigyl_Fwd	ACAGGCCTGCAAACGTTAAAA
Pigyl_Rev	CAGCGGTTACCAGCGACT
Rhox13_Fwd	CCCAGTACCCGGATTTGCTT
Rhox13_Rev	ACCACACCTTCACTTTGACCT
Tcfl5_Fwd	GAACGAGATAGGAGGCGCAG
Tcfl5_Rev	TCTCCGCATTGCAGAATGGA

Supplementary Table 2. List of GEO datasets used in this study.

	Cell type	GEO Accession	GEO Sample	Reference
**H3.3 ChIP-seq**	WT	GSE59271	GSM1432232	Voon *et al.* (*1*)
**KDM4a ChIP-seq**	WT	GSE64252	GSM2143305	Pedersen *et al.* (*2*)
**KDM4b ChIP-seq**	WT	GSE43231	GSM1058995	Das *et al.* (*3*)
**KDM4c ChIP-seq**	WT	GSE43231	GSM1058996	Das *et al.* (*3*)
**H3K9me3 ChIP-seq**	KDM4 A/B/C Flox Control	GSE64252	GSM2143309	Pedersen *et al.* (*2*)
**H3K9me3 ChIP-seq**	KDM4-tKO (+OHT)	GSE64252	GSM2143310	Pedersen *et al.* (*2*)
**H3K36me3 ChIP-seq**	KDM4 A/B/C Flox Control	GSE64252	GSM2143317	Pedersen *et al.* (*2*)
**H3K36me3 ChIP-seq**	KDM4-tKO (+OHT)	GSE64252	GSM2143318	Pedersen *et al.* (*2*)

Supplementary References

1. H. P. Voon, J. R. Hughes, C. Rode, I. A. De La Rosa-Velazquez, T. Jenuwein *et al.*, ATRX Plays a Key Role in Maintaining Silencing at Interstitial Heterochromatic Loci and Imprinted Genes. *Cell reports* **11**, 405-418 (2015).

2. M. T. Pedersen, S. M. Kooistra, A. Radzisheuskaya, A. Laugesen, J. V. Johansen *et al.*, Continual removal of H3K9 promoter methylation by Jmjd2 demethylases is vital for ESC self-renewal and early development. *The EMBO journal* **35**, 1550-1564 (2016).

3. P. P. Das, Z. Shao, S. Beyaz, E. Apostolou, L. Pinello *et al.*, Distinct and combinatorial functions of Jmjd2b/Kdm4b and Jmjd2c/Kdm4c in mouse embryonic stem cell identity. *Molecular cell* **53**, 32-48 (2014).
