# Supplementary material for: Inhibition of a K9/K36 demethylase by an H3.3 point mutation found in paediatric glioblastoma
Source: Nat Commun. 2018 Aug 7;9:3142. doi: 10.1038/s41467-018-05607-5 (PMC6081460; doi:10.1038/s41467-018-05607-5)
Supplement: Supplementary file 3 — Description of Additional Supplementary Files [file 41467_2018_5607_MOESM3_ESM.pdf]

Description of Additional Supplementary Files

File Name: Supplementary Data 1
Description: List of genes which are down- or up- regulated in H3.3 G34R mutants relative to WT.
